# Supplementary material for: Hot spot prediction in protein-protein interactions by an ensemble system
Source: BMC Syst Biol. 2018 Dec 31;12(Suppl 9):132. doi: 10.1186/s12918-018-0665-8 (PMC6311905; doi:10.1186/s12918-018-0665-8)
Supplement: Supplementary file 2 — All relevant MATLAB code. A simple Matlab implement of our predictor. (ZIP 19,742 kb) [file 12918_2018_665_MOESM2_ESM.zip › Additional file 2/Read ASA/ASA Property/SKEMPI/2PCCA.txt]

 NetSurfP - Protein Surface Accessibility and Secondary Structure Predictions

  

|  |  |  |
| --- | --- | --- |
|  |  | NetSurfP - Protein Surface Accessibility and Secondary Structure Predictions  Technical University of Denmark |

  


---

```
# For publication of results, please cite:
# A generic method for assignment of reliability scores applied to solvent accessibility predictions.
# Bent Petersen, Thomas Nordahl Petersen, Pernille Andersen, Morten Nielsen and Claus Lundegaard
# BMC Structural Biology 2009, 9:51 doi:10.1186/1472-6807-9-51
#
# Column 1: Class assignment - B for buried or E for Exposed - Threshold: 25% exposure, but not based on RSA
# Column 2: Amino acid
# Column 3: Sequence name
# Column 4: Amino acid number
# Column 5: Relative Surface Accessibility - RSA
# Column 6: Absolute Surface Accessibility
# Column 7: Z-fit score for RSA prediction
# Column 8: Probability for Alpha-Helix
# Column 9: Probability for Beta-strand
# Column 10: Probability for Coil
E T  2PCCA                  1    0.796 110.419  -1.405   0.003   0.003   0.994
E T  2PCCA                  2    0.427  59.169  -0.769   0.052   0.084   0.864
B P  2PCCA                  3    0.272  38.583  -1.153   0.118   0.150   0.732
B L  2PCCA                  4    0.220  40.374  -0.056   0.064   0.216   0.721
B V  2PCCA                  5    0.204  31.293  -1.458   0.066   0.296   0.638
E H  2PCCA                  6    0.392  71.287  -0.383   0.069   0.386   0.545
B V  2PCCA                  7    0.205  31.432  -0.317   0.066   0.296   0.638
B A  2PCCA                  8    0.202  22.293  -1.129   0.118   0.150   0.732
E S  2PCCA                  9    0.378  44.278  -0.547   0.191   0.086   0.723
B V  2PCCA                 10    0.226  34.721   0.358   0.113   0.087   0.800
E E  2PCCA                 11    0.438  76.449  -1.161   0.184   0.043   0.773
E K  2PCCA                 12    0.452  92.976  -1.361   0.115   0.016   0.868
E G  2PCCA                 13    0.318  25.034  -1.239   0.058   0.017   0.925
E R  2PCCA                 14    0.605 138.499   0.313   0.058   0.017   0.925
E S  2PCCA                 15    0.295  34.597  -0.420   0.058   0.017   0.925
B Y  2PCCA                 16    0.108  23.080   0.952   0.115   0.016   0.868
E E  2PCCA                 17    0.496  86.704  -0.401   0.246   0.004   0.750
E D  2PCCA                 18    0.453  65.335   0.914   0.406   0.004   0.590
B F  2PCCA                 19    0.038   7.586   0.285   0.970   0.001   0.030
E Q  2PCCA                 20    0.379  67.672   1.290   0.970   0.001   0.030
E K  2PCCA                 21    0.453  93.100   1.666   0.970   0.001   0.030
B V  2PCCA                 22    0.030   4.534   0.764   0.970   0.001   0.030
B Y  2PCCA                 23    0.079  16.968   1.330   0.970   0.001   0.030
E N  2PCCA                 24    0.488  71.458   1.886   0.970   0.001   0.030
E A  2PCCA                 25    0.327  36.090   1.572   0.970   0.001   0.030
B I  2PCCA                 26    0.020   3.645   1.298   0.988   0.000   0.012
B A  2PCCA                 27    0.138  15.208   1.240   0.988   0.000   0.012
E L  2PCCA                 28    0.505  92.539   1.969   0.970   0.001   0.030
B K  2PCCA                 29    0.128  26.350   0.976   0.970   0.001   0.030
B L  2PCCA                 30    0.038   7.013   0.243   0.923   0.002   0.076
E R  2PCCA                 31    0.393  89.974   1.115   0.858   0.002   0.139
E E  2PCCA                 32    0.541  94.583   0.392   0.694   0.003   0.303
B D  2PCCA                 33    0.224  32.206  -0.594   0.321   0.003   0.675
E D  2PCCA                 34    0.421  60.623  -0.776   0.321   0.003   0.675
E E  2PCCA                 35    0.538  94.058   0.434   0.339   0.016   0.645
B Y  2PCCA                 36    0.137  29.256  -0.115   0.430   0.016   0.555
E D  2PCCA                 37    0.390  56.257  -1.083   0.339   0.016   0.645
E N  2PCCA                 38    0.634  92.818  -1.234   0.257   0.016   0.727
B Y  2PCCA                 39    0.292  62.443  -0.553   0.181   0.016   0.803
B I  2PCCA                 40    0.230  42.476  -0.651   0.115   0.016   0.868
B G  2PCCA                 41    0.206  16.189  -1.109   0.113   0.043   0.844
B Y  2PCCA                 42    0.051  10.899   0.333   0.268   0.043   0.689
B G  2PCCA                 43    0.025   1.967   0.434   0.455   0.046   0.498
B P  2PCCA                 44    0.038   5.421  -0.446   0.660   0.049   0.291
B V  2PCCA                 45    0.055   8.423  -1.071   0.779   0.100   0.120
B L  2PCCA                 46    0.022   4.083   0.922   0.725   0.163   0.112
B V  2PCCA                 47    0.019   2.859   1.288   0.725   0.163   0.112
B R  2PCCA                 48    0.048  10.923   0.542   0.662   0.225   0.113
B L  2PCCA                 49    0.019   3.479   1.267   0.662   0.225   0.113
B A  2PCCA                 50    0.026   2.876   0.833   0.578   0.229   0.194
B W  2PCCA                 51    0.027   6.421   0.369   0.453   0.248   0.299
B H  2PCCA                 52    0.030   5.366   0.341   0.307   0.165   0.527
B I  2PCCA                 53    0.031   5.680  -0.489   0.278   0.093   0.628
B S  2PCCA                 54    0.015   1.781   1.099   0.199   0.152   0.649
B G  2PCCA                 55    0.034   2.660  -0.323   0.216   0.235   0.548
B T  2PCCA                 56    0.030   4.147  -0.477   0.135   0.317   0.548
B W  2PCCA                 57    0.151  36.436   0.295   0.056   0.142   0.802
B D  2PCCA                 58    0.210  30.304  -0.745   0.018   0.088   0.893
B K  2PCCA                 59    0.084  17.217  -0.975   0.018   0.088   0.893
B H  2PCCA                 60    0.248  45.057  -1.111   0.052   0.084   0.864
B D  2PCCA                 61    0.134  19.324  -1.182   0.018   0.047   0.935
B N  2PCCA                 62    0.132  19.369  -1.932   0.018   0.088   0.893
B T  2PCCA                 63    0.114  15.812  -0.607   0.021   0.279   0.699
B G  2PCCA                 64    0.057   4.447  -1.173   0.022   0.359   0.619
B G  2PCCA                 65    0.041   3.211  -1.372   0.021   0.279   0.699
B S  2PCCA                 66    0.142  16.596  -1.158   0.019   0.141   0.840
E Y  2PCCA                 67    0.364  77.787  -1.868   0.018   0.047   0.935
E G  2PCCA                 68    0.382  30.087  -1.927   0.018   0.019   0.964
B G  2PCCA                 69    0.165  12.986  -3.018   0.053   0.043   0.903
B T  2PCCA                 70    0.271  37.546  -1.493   0.053   0.043   0.903
E Y  2PCCA                 71    0.345  73.748  -1.764   0.053   0.043   0.903
E R  2PCCA                 72    0.340  77.860  -0.729   0.053   0.043   0.903
B F  2PCCA                 73    0.251  50.336  -1.891   0.113   0.043   0.844
E K  2PCCA                 74    0.405  83.350  -0.451   0.184   0.043   0.773
E K  2PCCA                 75    0.234  48.072  -1.197   0.268   0.043   0.689
B E  2PCCA                 76    0.045   7.809  -3.350   0.268   0.043   0.689
B F  2PCCA                 77    0.255  51.219  -0.580   0.184   0.043   0.773
E N  2PCCA                 78    0.379  55.500  -1.123   0.115   0.016   0.868
B D  2PCCA                 79    0.217  31.327  -0.606   0.058   0.017   0.925
E P  2PCCA                 80    0.331  46.941  -0.970   0.115   0.016   0.868
E S  2PCCA                 81    0.418  48.990  -0.621   0.058   0.017   0.925
B N  2PCCA                 82    0.081  11.814  -3.440   0.115   0.016   0.868
E A  2PCCA                 83    0.318  35.011  -0.292   0.115   0.016   0.868
B G  2PCCA                 84    0.228  17.920  -1.334   0.115   0.016   0.868
B L  2PCCA                 85    0.057  10.345   0.157   0.600   0.003   0.397
E Q  2PCCA                 86    0.461  82.388   0.629   0.858   0.002   0.139
E N  2PCCA                 87    0.320  46.892   1.407   0.858   0.002   0.139
B G  2PCCA                 88    0.014   1.133   0.877   0.970   0.001   0.030
B F  2PCCA                 89    0.123  24.706   0.776   0.970   0.001   0.030
E K  2PCCA                 90    0.469  96.473   1.893   0.970   0.001   0.030
B F  2PCCA                 91    0.042   8.389   0.742   0.970   0.001   0.030
B L  2PCCA                 92    0.027   5.017   0.766   0.970   0.001   0.030
E E  2PCCA                 93    0.330  57.616   1.108   0.970   0.001   0.030
E P  2PCCA                 94    0.370  52.531   1.180   0.923   0.002   0.076
B I  2PCCA                 95    0.025   4.588   0.665   0.923   0.002   0.076
E H  2PCCA                 96    0.254  46.130   0.449   0.858   0.002   0.139
E K  2PCCA                 97    0.629 129.468   0.501   0.858   0.002   0.139
E E  2PCCA                 98    0.344  60.167   0.659   0.694   0.003   0.303
B F  2PCCA                 99    0.147  29.503   0.882   0.321   0.003   0.675
E P  2PCCA                100    0.561  79.648  -0.890   0.053   0.005   0.942
E W  2PCCA                101    0.454 109.187  -0.553   0.018   0.019   0.964
B I  2PCCA                102    0.115  21.368   0.160   0.058   0.017   0.925
B S  2PCCA                103    0.117  13.689  -0.942   0.115   0.016   0.868
B S  2PCCA                104    0.092  10.759   0.492   0.879   0.010   0.111
B G  2PCCA                105    0.115   9.074  -0.534   0.938   0.007   0.055
B D  2PCCA                106    0.117  16.932  -0.296   0.938   0.007   0.055
B L  2PCCA                107    0.022   4.028   1.000   0.938   0.007   0.055
B F  2PCCA                108    0.033   6.683   0.247   0.938   0.007   0.055
B S  2PCCA                109    0.052   6.141  -0.166   0.938   0.007   0.055
B L  2PCCA                110    0.052   9.521   0.084   0.938   0.007   0.055
B G  2PCCA                111    0.022   1.692   0.497   0.879   0.010   0.111
B G  2PCCA                112    0.031   2.424  -0.275   0.879   0.010   0.111
B V  2PCCA                113    0.113  17.337  -0.455   0.938   0.007   0.055
B T  2PCCA                114    0.112  15.604   0.176   0.923   0.002   0.076
B A  2PCCA                115    0.015   1.642   1.099   0.975   0.003   0.022
B V  2PCCA                116    0.035   5.426  -0.254   0.975   0.003   0.022
E Q  2PCCA                117    0.223  39.846   0.429   0.938   0.007   0.055
B E  2PCCA                118    0.205  35.744  -0.633   0.879   0.010   0.111
B M  2PCCA                119    0.059  11.846  -0.655   0.522   0.016   0.462
E Q  2PCCA                120    0.300  53.598  -1.926   0.053   0.005   0.942
B G  2PCCA                121    0.196  15.433  -1.374   0.003   0.003   0.994
B P  2PCCA                122    0.282  40.030  -0.467   0.005   0.045   0.951
E K  2PCCA                123    0.404  83.123  -1.061   0.005   0.336   0.660
B I  2PCCA                124    0.096  17.760   0.389   0.004   0.420   0.576
E P  2PCCA                125    0.466  66.083  -0.494   0.022   0.359   0.619
B W  2PCCA                126    0.263  63.227  -0.116   0.019   0.141   0.840
E R  2PCCA                127    0.358  81.936  -1.056   0.052   0.084   0.864
B C  2PCCA                128    0.180  25.258  -0.594   0.018   0.047   0.935
B G  2PCCA                129    0.072   5.706  -2.473   0.018   0.047   0.935
B R  2PCCA                130    0.136  31.258  -0.307   0.018   0.088   0.893
E V  2PCCA                131    0.397  60.988   0.054   0.052   0.084   0.864
B D  2PCCA                132    0.188  27.019  -1.158   0.053   0.043   0.903
B T  2PCCA                133    0.159  21.998  -0.944   0.184   0.043   0.773
E P  2PCCA                134    0.379  53.766  -0.698   0.184   0.043   0.773
E E  2PCCA                135    0.347  60.691  -0.534   0.268   0.043   0.689
B D  2PCCA                136    0.262  37.797  -2.538   0.184   0.043   0.773
E T  2PCCA                137    0.356  49.419  -0.528   0.184   0.043   0.773
B T  2PCCA                138    0.249  34.522  -1.549   0.268   0.043   0.689
E P  2PCCA                139    0.373  52.872  -0.041   0.257   0.016   0.727
E D  2PCCA                140    0.570  82.166  -1.546   0.257   0.016   0.727
E N  2PCCA                141    0.425  62.161  -1.218   0.181   0.016   0.803
E G  2PCCA                142    0.652  51.281  -2.339   0.058   0.017   0.925
E R  2PCCA                143    0.333  76.188  -1.182   0.018   0.019   0.964
B L  2PCCA                144    0.114  20.928  -0.044   0.018   0.019   0.964
B P  2PCCA                145    0.081  11.451  -1.837   0.005   0.015   0.979
E D  2PCCA                146    0.306  44.138  -0.313   0.005   0.015   0.979
B A  2PCCA                147    0.177  19.461  -1.794   0.005   0.015   0.979
E D  2PCCA                148    0.485  69.961  -1.155   0.018   0.019   0.964
B K  2PCCA                149    0.244  50.150  -1.246   0.018   0.019   0.964
E D  2PCCA                150    0.402  57.856   0.158   0.053   0.005   0.942
B A  2PCCA                151    0.067   7.416  -0.418   0.923   0.002   0.076
E G  2PCCA                152    0.570  44.843   1.244   0.923   0.002   0.076
E Y  2PCCA                153    0.379  80.950   1.221   0.923   0.002   0.076
B V  2PCCA                154    0.035   5.441   0.256   0.970   0.001   0.030
B R  2PCCA                155    0.205  47.059   1.367   0.970   0.001   0.030
E T  2PCCA                156    0.502  69.614   1.023   0.970   0.001   0.030
B F  2PCCA                157    0.164  32.915   1.487   0.970   0.001   0.030
B F  2PCCA                158    0.033   6.563  -0.707   0.970   0.001   0.030
E Q  2PCCA                159    0.377  67.368   0.533   0.923   0.002   0.076
E R  2PCCA                160    0.654 149.743  -0.099   0.782   0.003   0.216
E L  2PCCA                161    0.287  52.550   0.759   0.246   0.004   0.750
E N  2PCCA                162    0.579  84.810  -0.559   0.016   0.005   0.979
B M  2PCCA                163    0.084  16.748  -0.502   0.003   0.003   0.994
E N  2PCCA                164    0.394  57.755   0.648   0.003   0.003   0.994
E D  2PCCA                165    0.378  54.455  -0.212   0.268   0.043   0.689
E R  2PCCA                166    0.404  92.447   0.767   0.268   0.043   0.689
B E  2PCCA                167    0.115  20.073  -0.036   0.199   0.152   0.649
B V  2PCCA                168    0.035   5.364   0.816   0.268   0.505   0.227
B V  2PCCA                169    0.018   2.813   1.164   0.085   0.775   0.140
B A  2PCCA                170    0.031   3.471   0.342   0.072   0.855   0.073
B L  2PCCA                171    0.017   3.113   1.240   0.072   0.855   0.073
B M  2PCCA                172    0.018   3.662   1.315   0.085   0.775   0.140
B G  2PCCA                173    0.036   2.849  -0.944   0.074   0.484   0.442
B A  2PCCA                174    0.035   3.901  -2.015   0.064   0.216   0.721
B H  2PCCA                175    0.053   9.677  -0.305   0.066   0.296   0.638
B A  2PCCA                176    0.031   3.416  -1.399   0.021   0.279   0.699
B L  2PCCA                177    0.058  10.528   0.190   0.066   0.296   0.638
B G  2PCCA                178    0.056   4.431  -2.262   0.019   0.141   0.840
E K  2PCCA                179    0.260  53.461  -0.494   0.019   0.141   0.840
B T  2PCCA                180    0.045   6.200  -2.231   0.056   0.142   0.802
B H  2PCCA                181    0.121  22.028  -0.494   0.020   0.205   0.775
B L  2PCCA                182    0.101  18.456  -0.641   0.020   0.205   0.775
B K  2PCCA                183    0.198  40.626  -0.897   0.019   0.141   0.840
B N  2PCCA                184    0.192  28.123  -1.832   0.018   0.088   0.893
B S  2PCCA                185    0.150  17.568  -2.540   0.018   0.047   0.935
B G  2PCCA                186    0.203  15.952  -2.689   0.018   0.088   0.893
B Y  2PCCA                187    0.225  48.061  -1.649   0.019   0.141   0.840
E E  2PCCA                188    0.341  59.503  -1.421   0.019   0.141   0.840
B G  2PCCA                189    0.285  22.453  -1.205   0.019   0.141   0.840
E P  2PCCA                190    0.356  50.559  -2.005   0.020   0.205   0.775
E W  2PCCA                191    0.361  86.748  -1.855   0.018   0.088   0.893
E G  2PCCA                192    0.457  35.934  -1.604   0.018   0.047   0.935
E A  2PCCA                193    0.585  64.456  -2.346   0.018   0.019   0.964
E A  2PCCA                194    0.549  60.544  -1.420   0.016   0.005   0.979
B N  2PCCA                195    0.257  37.566  -1.659   0.016   0.005   0.979
B N  2PCCA                196    0.289  42.339  -1.486   0.016   0.005   0.979
E V  2PCCA                197    0.315  48.385  -1.145   0.058   0.017   0.925
B F  2PCCA                198    0.075  15.153  -0.827   0.058   0.017   0.925
B T  2PCCA                199    0.168  23.343  -0.888   0.018   0.019   0.964
B N  2PCCA                200    0.289  42.310  -1.021   0.694   0.003   0.303
E E  2PCCA                201    0.440  76.903  -1.192   0.782   0.003   0.216
B F  2PCCA                202    0.081  16.217  -0.439   0.858   0.002   0.139
B Y  2PCCA                203    0.029   6.090  -0.415   0.923   0.002   0.076
E L  2PCCA                204    0.303  55.479   0.642   0.970   0.001   0.030
E N  2PCCA                205    0.257  37.581   0.102   0.923   0.002   0.076
B L  2PCCA                206    0.035   6.390  -1.071   0.858   0.002   0.139
B L  2PCCA                207    0.140  25.652   0.008   0.782   0.003   0.216
E N  2PCCA                208    0.621  90.929   0.174   0.502   0.002   0.495
E E  2PCCA                209    0.398  69.583  -0.454   0.176   0.004   0.820
E D  2PCCA                210    0.298  42.899  -1.566   0.058   0.017   0.925
B W  2PCCA                211    0.124  29.774  -1.020   0.018   0.047   0.935
E K  2PCCA                212    0.580 119.347  -1.075   0.018   0.047   0.935
E L  2PCCA                213    0.311  56.853  -0.759   0.018   0.047   0.935
E E  2PCCA                214    0.452  79.034  -1.638   0.018   0.019   0.964
E K  2PCCA                215    0.533 109.679  -1.918   0.018   0.019   0.964
E N  2PCCA                216    0.412  60.361  -2.011   0.018   0.019   0.964
E D  2PCCA                217    0.497  71.618  -2.095   0.018   0.019   0.964
E A  2PCCA                218    0.434  47.882  -2.393   0.018   0.019   0.964
E N  2PCCA                219    0.405  59.321  -0.658   0.018   0.047   0.935
B N  2PCCA                220    0.229  33.599  -1.555   0.020   0.205   0.775
B E  2PCCA                221    0.245  42.767   0.221   0.022   0.552   0.426
B Q  2PCCA                222    0.131  23.325  -0.554   0.023   0.655   0.322
B W  2PCCA                223    0.152  36.484   0.030   0.023   0.655   0.322
E D  2PCCA                224    0.241  34.670  -0.052   0.021   0.451   0.528
B S  2PCCA                225    0.228  26.745  -0.348   0.066   0.296   0.638
E K  2PCCA                226    0.374  76.911  -0.250   0.056   0.142   0.802
E S  2PCCA                227    0.556  65.222  -0.367   0.052   0.084   0.864
E G  2PCCA                228    0.480  37.752  -1.065   0.053   0.043   0.903
E Y  2PCCA                229    0.283  60.456   0.261   0.064   0.216   0.721
B M  2PCCA                230    0.065  13.067  -1.364   0.022   0.552   0.426
B M  2PCCA                231    0.107  21.411   0.256   0.023   0.655   0.322
B L  2PCCA                232    0.044   8.001   0.408   0.079   0.592   0.329
E P  2PCCA                233    0.242  34.297  -0.015   0.231   0.330   0.439
B T  2PCCA                234    0.036   4.965  -0.300   0.605   0.105   0.290
B D  2PCCA                235    0.053   7.637  -0.702   0.717   0.014   0.269
E Y  2PCCA                236    0.242  51.630   0.316   0.802   0.014   0.185
B S  2PCCA                237    0.149  17.510   0.095   0.802   0.014   0.185
B L  2PCCA                238    0.041   7.452   0.465   0.717   0.014   0.269
B I  2PCCA                239    0.277  51.245  -0.275   0.522   0.016   0.462
E Q  2PCCA                240    0.507  90.604   0.169   0.181   0.016   0.803
E D  2PCCA                241    0.266  38.302   0.262   0.018   0.019   0.964
E P  2PCCA                242    0.667  94.690  -1.185   0.321   0.003   0.675
E K  2PCCA                243    0.499 102.706   0.675   0.600   0.003   0.397
B Y  2PCCA                244    0.042   8.954  -0.218   0.858   0.002   0.139
B L  2PCCA                245    0.213  39.092   1.039   0.970   0.001   0.030
E S  2PCCA                246    0.493  57.826   1.197   0.970   0.001   0.030
B I  2PCCA                247    0.102  18.870   1.455   0.970   0.001   0.030
B V  2PCCA                248    0.015   2.321   1.458   0.988   0.000   0.012
E K  2PCCA                249    0.376  77.425   1.731   0.970   0.001   0.030
E E  2PCCA                250    0.386  67.434   1.521   0.975   0.003   0.022
B Y  2PCCA                251    0.064  13.570   0.613   0.938   0.007   0.055
B A  2PCCA                252    0.149  16.409  -0.840   0.694   0.003   0.303
E N  2PCCA                253    0.624  91.339   0.471   0.321   0.003   0.675
E D  2PCCA                254    0.354  51.055   0.075   0.016   0.005   0.979
E Q  2PCCA                255    0.315  56.205   0.268   0.694   0.003   0.303
E D  2PCCA                256    0.619  89.183   0.906   0.923   0.002   0.076
E K  2PCCA                257    0.343  70.576   2.131   0.970   0.001   0.030
B F  2PCCA                258    0.034   6.844   0.551   0.970   0.001   0.030
B F  2PCCA                259    0.163  32.714   0.700   0.970   0.001   0.030
E K  2PCCA                260    0.517 106.306   1.271   0.970   0.001   0.030
B D  2PCCA                261    0.224  32.250   1.523   0.988   0.000   0.012
B F  2PCCA                262    0.030   6.061   0.946   0.988   0.000   0.012
B S  2PCCA                263    0.066   7.747   0.263   0.988   0.000   0.012
E K  2PCCA                264    0.382  78.557   1.892   0.988   0.000   0.012
B A  2PCCA                265    0.030   3.328   0.300   0.988   0.000   0.012
B F  2PCCA                266    0.053  10.617   0.422   0.988   0.000   0.012
B E  2PCCA                267    0.169  29.489   0.199   0.970   0.001   0.030
B K  2PCCA                268    0.197  40.605   1.013   0.938   0.007   0.055
B L  2PCCA                269    0.041   7.452  -0.133   0.879   0.010   0.111
B L  2PCCA                270    0.110  20.178  -1.237   0.717   0.014   0.269
E E  2PCCA                271    0.476  83.122  -0.373   0.268   0.043   0.689
B N  2PCCA                272    0.093  13.586   0.446   0.052   0.084   0.864
E G  2PCCA                273    0.459  36.139  -0.791   0.018   0.047   0.935
B I  2PCCA                274    0.085  15.651  -0.320   0.018   0.047   0.935
E T  2PCCA                275    0.326  45.202  -0.447   0.018   0.047   0.935
E F  2PCCA                276    0.338  67.897  -0.793   0.018   0.019   0.964
E P  2PCCA                277    0.283  40.229  -2.082   0.018   0.019   0.964
E K  2PCCA                278    0.573 117.866  -1.741   0.018   0.019   0.964
E D  2PCCA                279    0.611  88.103  -0.344   0.018   0.019   0.964
B A  2PCCA                280    0.225  24.806  -1.500   0.018   0.088   0.893
E P  2PCCA                281    0.461  65.373  -0.170   0.020   0.205   0.775
E S  2PCCA                282    0.292  34.187  -0.535   0.021   0.279   0.699
E P  2PCCA                283    0.308  43.634  -0.593   0.021   0.279   0.699
E F  2PCCA                284    0.302  60.692  -0.084   0.022   0.359   0.619
E I  2PCCA                285    0.385  71.151   0.248   0.022   0.359   0.619
B F  2PCCA                286    0.099  19.930   0.545   0.020   0.205   0.775
E K  2PCCA                287    0.466  95.918   0.463   0.020   0.205   0.775
E T  2PCCA                288    0.434  60.168   0.509   0.019   0.141   0.840
B L  2PCCA                289    0.170  31.182   0.794   0.018   0.088   0.893
E E  2PCCA                290    0.428  74.684  -0.102   0.018   0.047   0.935
E E  2PCCA                291    0.595 104.016  -0.596   0.018   0.019   0.964
E Q  2PCCA                292    0.644 114.965  -0.773   0.018   0.019   0.964
E G  2PCCA                293    0.622  48.944  -1.823   0.018   0.019   0.964
E L  2PCCA                294    0.504  92.282  -1.901   0.003   0.003   0.994

Explain the output.  Go back.
```

---
